# Supplementary material for: Further evidence that CP-AMPARs are critically involved in synaptic tag and capture at hippocampal CA1 synapses
Source: Mol Brain. 2021 Feb 1;14:26. doi: 10.1186/s13041-021-00737-2 (PMC7851922; doi:10.1186/s13041-021-00737-2)
Supplement: Supplementary file 1 — Additional file 1: Figure 1. Hypothetical scheme for heterosynaptic metaplasticity. [file 13041_2021_737_MOESM1_ESM.docx]

**Further evidence that CP-AMPARs are critically involved in synaptic tag and capture at hippocampal CA1 synapses**

Pojeong Park^1,2,3,4^, Heather Kang^2,3,4^, John Georgiou^3^, Min Zhuo^1,2^, Bong-Kiun Kaang^1^ and Graham L. Collingridge^1,2,3,4,5,*^

**Additional information**


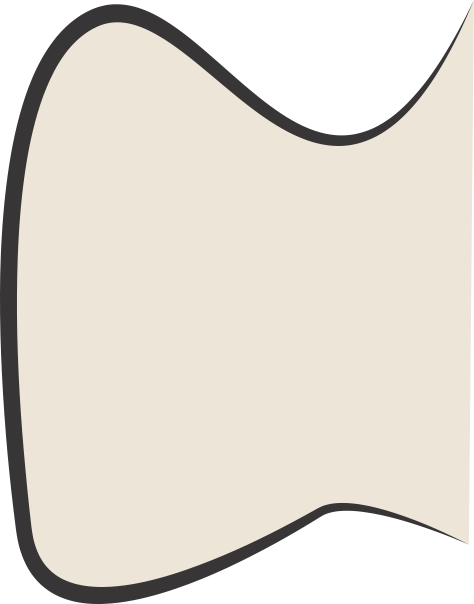

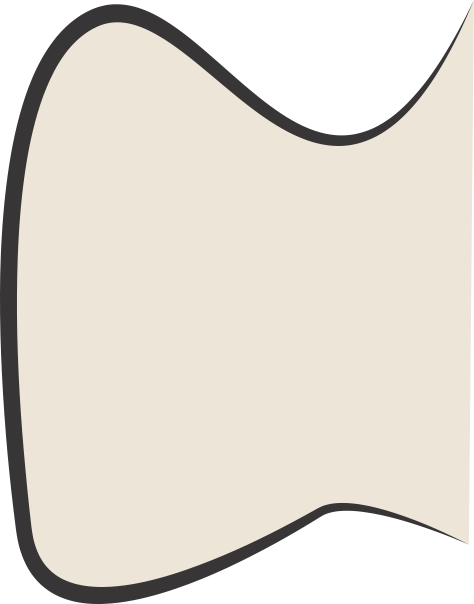

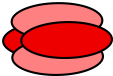

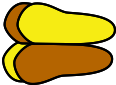

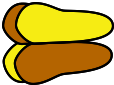

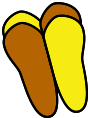

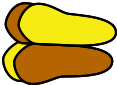

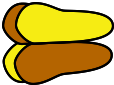

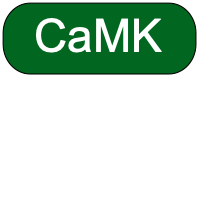

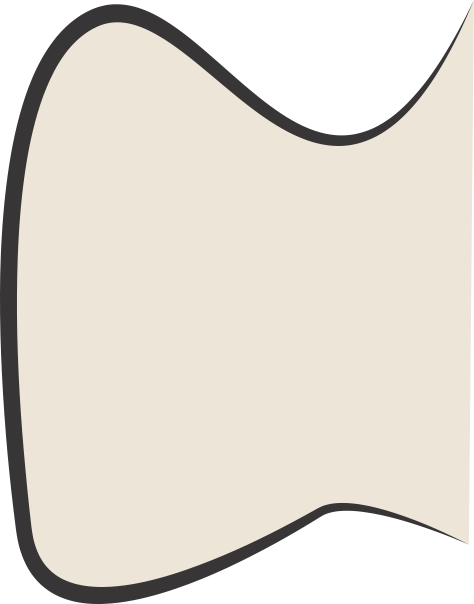

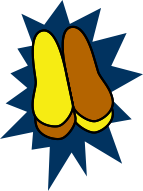

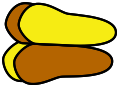

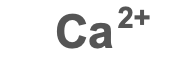

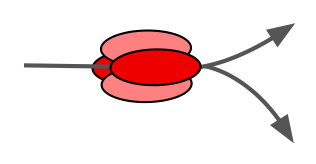

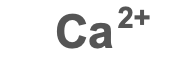

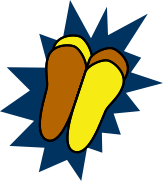

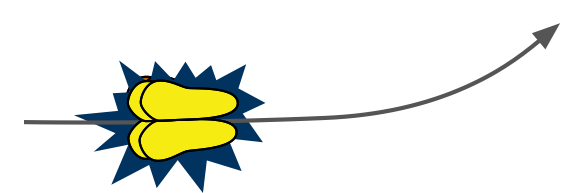

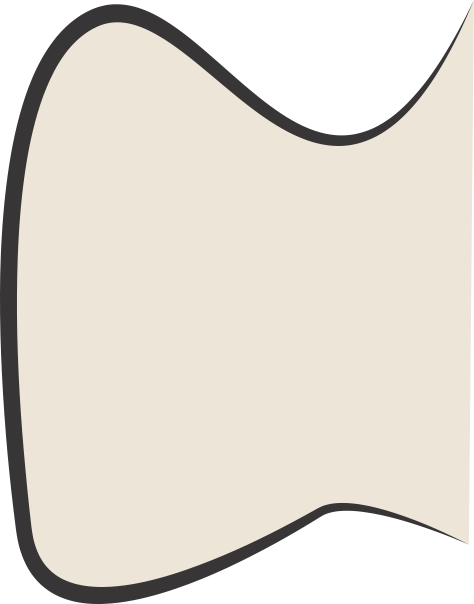

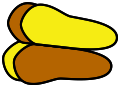

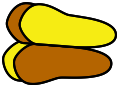

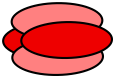

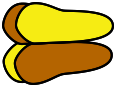

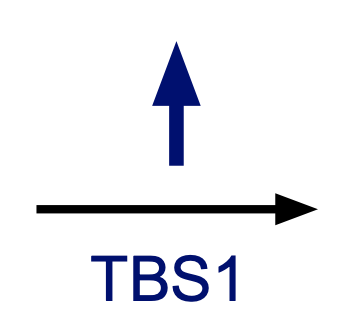

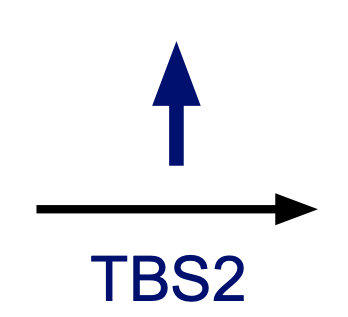

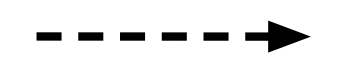

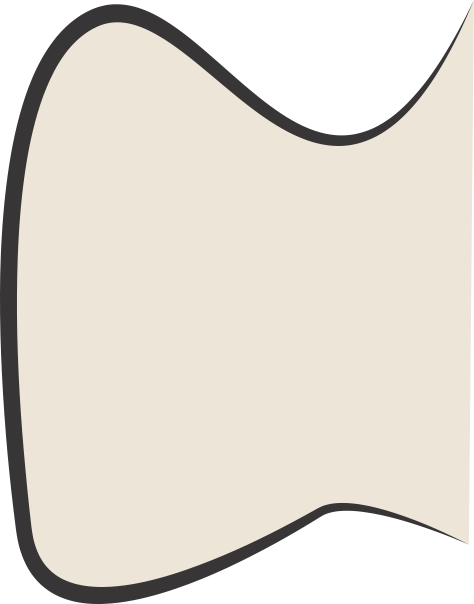

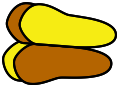

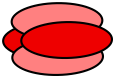

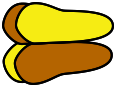

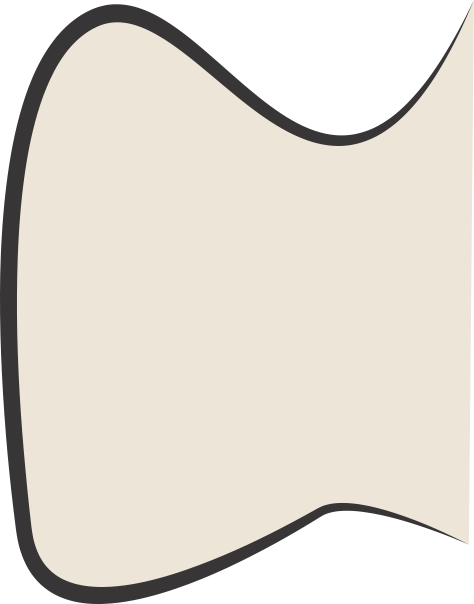

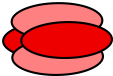

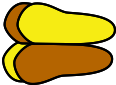

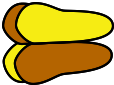

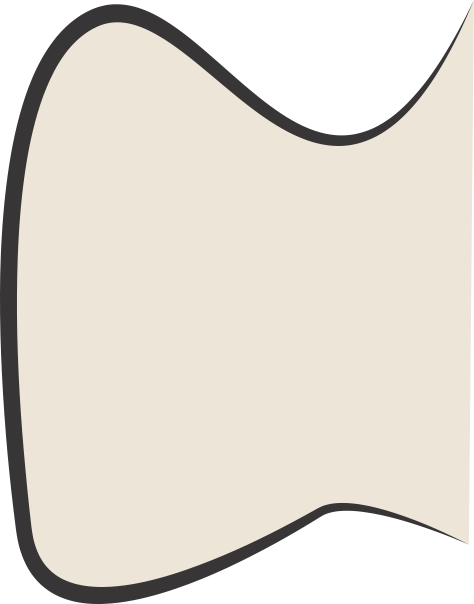

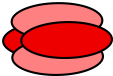

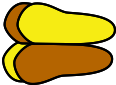

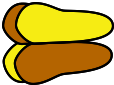

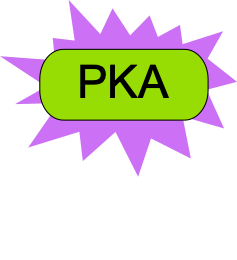

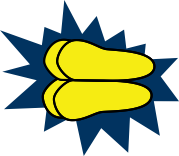

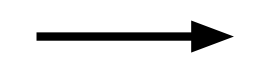

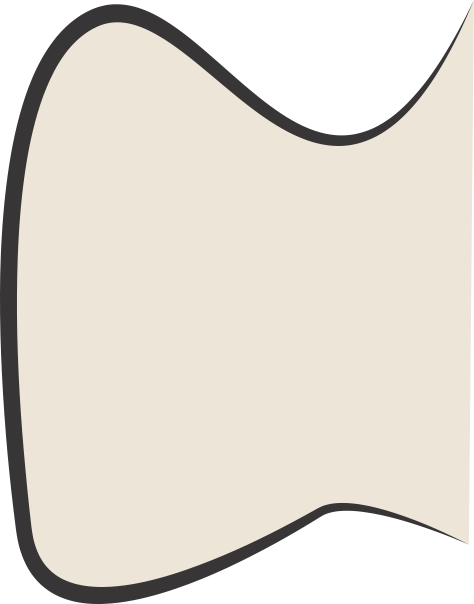

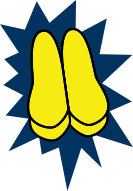

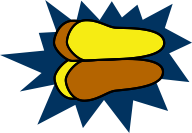

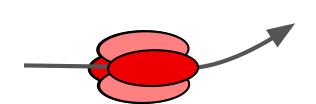

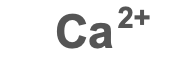

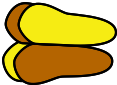

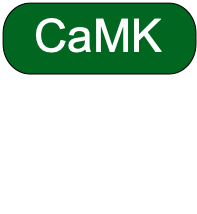

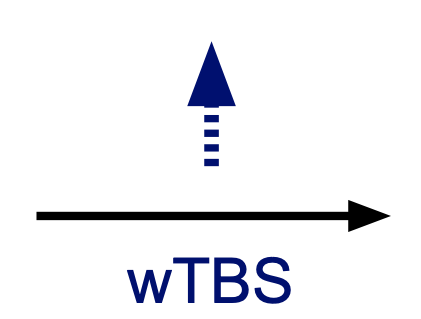

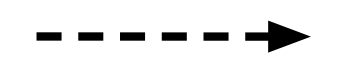

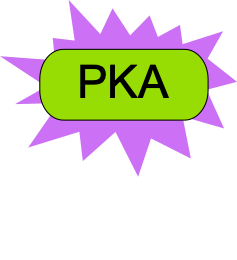

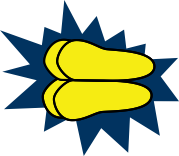

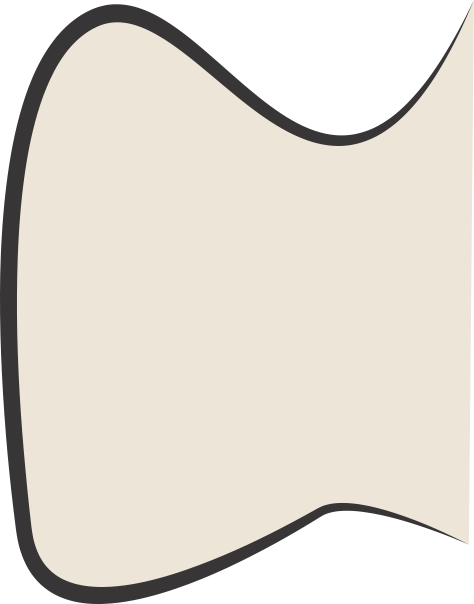

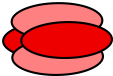

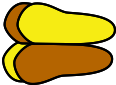

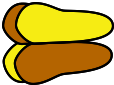

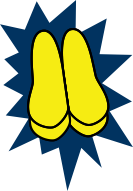


**“Tagged”**


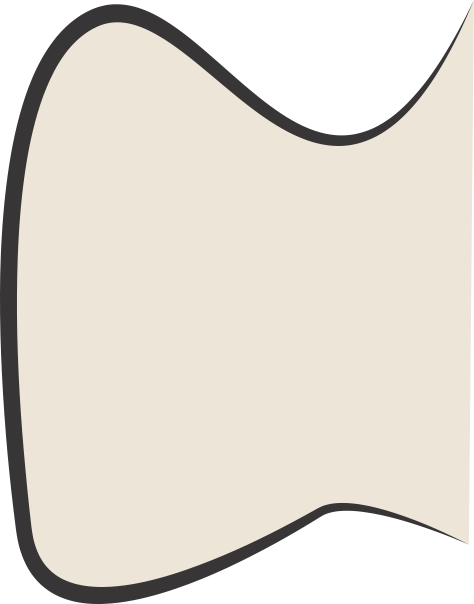

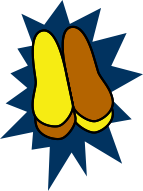

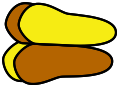

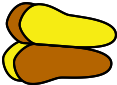

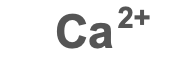

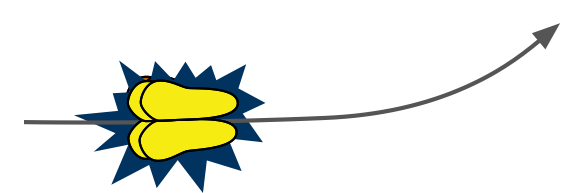

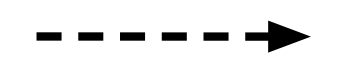

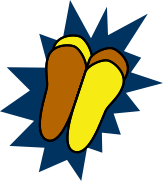

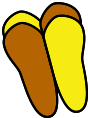

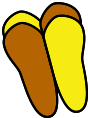

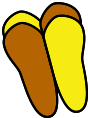

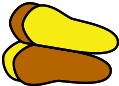

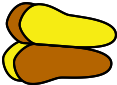


***LTP1***

***LTP2***


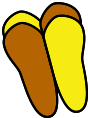

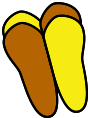

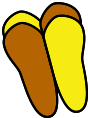

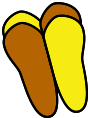

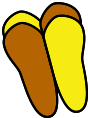

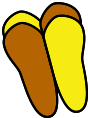

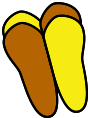

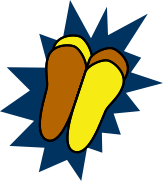

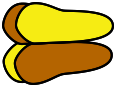

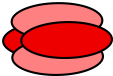

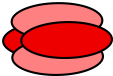

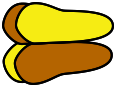


***Strong stim***

***Weak stim***

**Baseline**

**Induction**

***LTP1***

***LTP2***

***Basal stim***

***Basal stim***


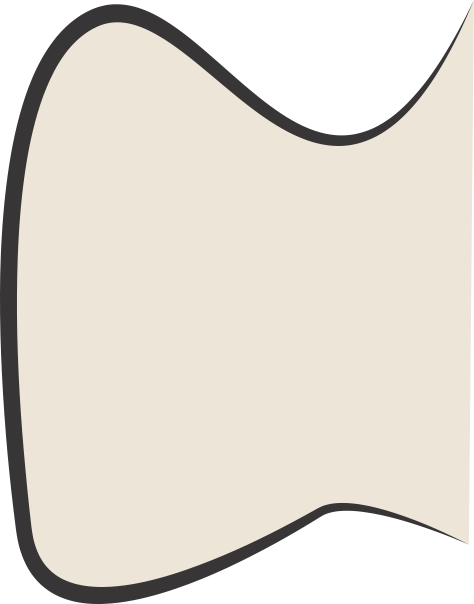

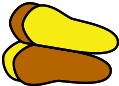

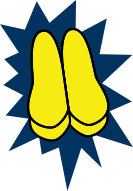

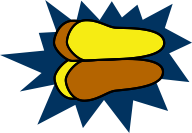

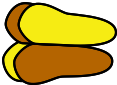

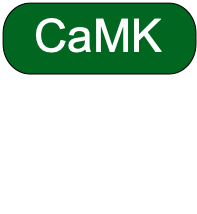

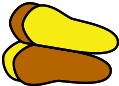

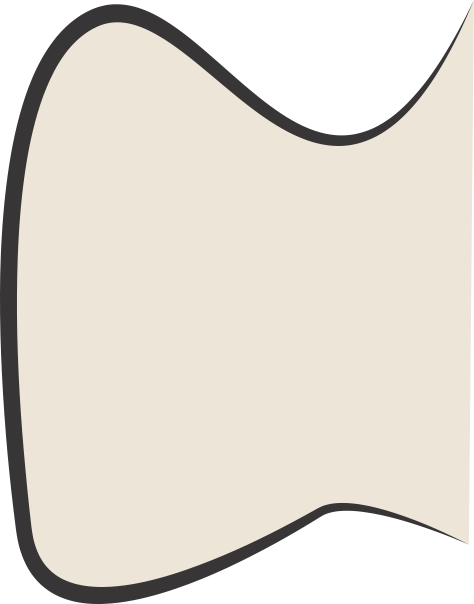

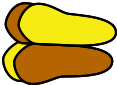

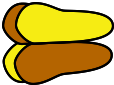

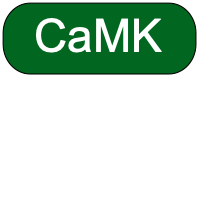

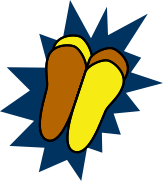

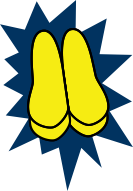


**Primed**


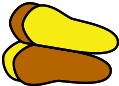

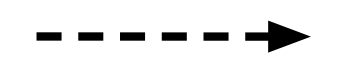


**cAMP**


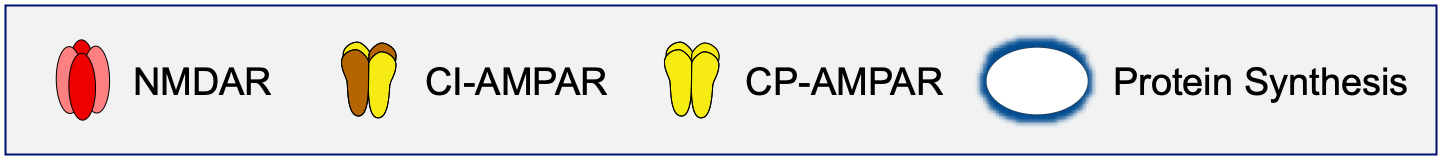

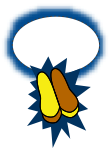

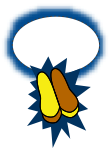

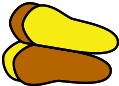


***Input 1***

***Input 2***

**Expression**

**Additional file 1: Figure 1.** Hypothetical scheme for heterosynaptic metaplasticity.

Two inputs (shown in rows) are depicted by individual spine targets. The inputs are independent as defined by the lack of heterosynaptic paired-pulse facilitation. A strong stimulus comprised of spaced TBS (sTBS) is delivered to the upper input and a weak TBS (wTBS) stimulus (comprised of a short, single TBS) is delivered to the lower input, 30 min later. The first TBS, within the sTBS, activates NMDA receptors (NMDARs) to trigger homosynaptic LTP1 via engaging CaMKII to drive calcium-impermeable AMPA receptors (CI-AMPARs) into the synapse. Simultaneously it primes for LTP2 by activating PKA, via the formation of cAMP, and this drives calcium-permeable AMPA receptors (CP-AMPARs) onto the plasma membrane at perisynaptic sites. The subsequent TBS2 (and TBS3, not shown) drives these CP-AMPARs into the synapse where they increase the response amplitude by virtue of their higher single channel conductance. Basal stimulation activates these CP-AMPARs to drive *de novo* protein synthesis resulting in the insertion of additional CI-AMPARs at the expense of the transiently expressed CP-AMPARs. The cAMP diffuses to adjacent synapses where it activates PKA to drive CP-AMPARs into the plasma membrane. Some make it to the synapse where they trigger heterosynaptic LTP and may be activated in response to a wTBS to trigger LTP2 (not illustrated – see Park et al, 2019). Others are restricted to perisynaptic sites, where they “tag” these surround synapses for LTP2. A weak TBS delivered during the dwell time that these CP-AMPARs are present perisynaptically drives these CP-AMPARs into the synapse. Basal stimulation activates these CP-AMPARs to trigger local *de novo* protein synthesis and the generation of LTP2.
